# A Prospective Study of Rumination and Irritability in Youth

**DOI:** 10.1007/s10802-020-00706-8

**Published:** 2020-10-01

**Authors:** Eleanor Leigh, Ailsa Lee, Hannah M. Brown, Simone Pisano, Argyris Stringaris

**Affiliations:** 1 Department of Experimental Psychology, University of Oxford, Oxford, UK; 2 OxCADAT, The Old Rectory, Paradise Square, Oxford, OX1 1TW UK; 3 Department of Psychology, Institute of Psychiatry, Psychology & Neuroscience, King’s College London, London, UK; 4 Department of Neuroscience, AORN Santobono-Pausilipon, Naples, Italy; 5 Department of Translational Medical Sciences, Federico II University, Naples, Italy; 6 Mood Brain and Development Unit, Emotion and Development Branch, National Institute of Mental Health, National Institutes of Health, Department of Health and Human Services, Bethesda, MD USA

**Keywords:** Adolescence, Irritability, Rumination, Angry rumination

## Abstract

Although youth irritability is linked with substantial psychiatric morbidity and impairment, little is known about how personal characteristics influence its course. In this study we examined the prospective associations between angry and depressive rumination and irritability. A sample of 165 school pupils aged 12–14 years were assessed at two time points six months apart. They completed measures of irritability at Times 1 and 2 and depressive and angry rumination at Time 1. In line with our hypotheses, we found that angry rumination is significantly associated with irritability six months later, over and above baseline irritability and depressive rumination. The present findings suggest angry rumination is relevant to the genesis of irritability in adolescents, and point to possible routes for prevention and early intervention.

## Introduction

Irritability in youth is one of the most common reasons for referral to mental health services, a predictor of future depression and suicidality, and associated with role impairment (Stringaris et al. 2018). Yet, it remains to be understood how person-specific characteristics contribute to variation in irritability. We test the hypothesis that increases in adolescent irritability are predicted by the tendency to engage in angry rumination.

Irritability, defined as individual differences in proneness to anger (Vidal-Ribas et al. 2016) and a reaction to blocked goal attainment (RDoC, Insel et al. (2010)), has been the focus of increasing research interest. Paediatric irritability has been linked to the development of a range of internalising disorders, and depression in particular (Stringaris et al. 2009; Stringaris and Goodman 2009; Leibenluft et al. 2006; Krieger et al. 2013). Understanding factors that contribute to the development of irritability may therefore provide opportunity for early prevention and intervention.

Person-specific characteristics are likely to contribute to irritability in adolescents. One such candidate characteristic is rumination. Rumination is a repetitive, negative thinking process, that is passive and internally-focused (e.g. Nolen-Hoeksema et al. (1993)), amplifying current mood states and impairing instrumental behaviour and problem-solving (Nolen-Hoeksema et al. 2008). Whilst first invoked as a risk factor for depression, rumination has since been established as a transdiagnostic risk factor across the lifespan (Aldao et al. 2010; Rood et al. 2009; Watkins 2008). This has led to the development of psychological interventions targeting rumination in order to prevent or reduce various forms of psychopathology, including adolescent depression and anxiety (for example, Jacobs et al. (2016), Topper et al. (2017)).

There have been far fewer studies examining angry rumination and its association with irritability and anger. Angry rumination is understood to be prompted when an individual’s goals are frustrated (Martin and Tesser 1996), as they seek to understand the causes and consequences of the disappointment and dwell on the feeling of anger. Angry rumination has been found to be associated with anger in experimental (Bushman 2002; Denson et al. 2012; Gerin et al. 2006; Rusting and Nolen-Hoeksema 1998) and cross-sectional (Sukhodolsky et al. 2001) studies. It has also been shown to be associated with aggressive behaviours in community samples of children, adolescents and adults (Bushman 2002; Harmon et al. 2019; Smith et al. 2016; Sukhodolsky et al. 2001). Two studies have shown that this association persists when controlling for trait anger (Peled and Moretti 2007, 2010), although this was not replicated in a third study with a sample of juvenile offenders (Smith et al. 2016). Whilst these findings are broadly consistent with the proposal that angry rumination predicts angry feelings and its behavioural correlates, only one of the studies (Smith et al. 2016) utilised a prospective design, which is necessary in order to determine the temporal ordering of the association.

A small number of studies have included measures of both depressive and angry rumination, which allows examination of the question of whether these two forms of rumination are better conceptualised as a unitary factor or distinct constructs. Whilst it has been reported that individuals who tend to engage in angry rumination are also more likely to engage in depressive rumination (Peled and Moretti 2007, 2010), a factor analytic study with a sample of clinic referred youth indicated that these two forms of rumination reflect two distinct factors (Peled and Moretti 2007). Furthermore, differential patterns of association between the two forms of rumination and depression, anger, and aggression have been observed. For example, a study by Gilbert and colleagues with adults (Gilbert et al. 2005) demonstrated that depressive, but not angry, rumination was associated with depression symptoms, however no measure of irritability or aggression was included. Studies with unselected adults (Peled and Moretti 2010), clinically referred adolescents (Peled and Moretti 2007), and unselected pre-adolescent children (Harmon et al. 2019) have found that angry, but not depressive, rumination is associated with feelings of anger and aggression, after controlling for shared variance. However, as yet no longitudinal study has been undertaken to examine the prospective association between angry rumination and irritability, over and above depressive rumination. This will be important to establish in order to determine whether angry rumination specifically, rather than rumination generally, should be targeted in strategies to prevent and treat problematic irritability.

### The Current Study

In order to better understand risk factors for youth irritability, and angry rumination in particular, we undertook a prospective questionnaire-based study with a community sample of British adolescents assessed two times over a six-month period. We focused on an adolescent sample for two reasons. First, irritability at this stage of life predicts important negative outcomes in adulthood (including reduced income and educational attainment; Stringaris et al. (2009)), and so establishing risk factors for irritability may provide opportunities for early intervention. Second, the shift from childhood to adolescence sees an increase in the use of rumination as a coping strategy in response to stress. For example, developmental increases in rumination were observed across a large sample of German youth aged 8 to 13 stratified by age (Hampel and Petermann 2005). We measured angry and depressive rumination at baseline and monitored change in irritability over time. It was hypothesised that, after controlling for baseline levels of irritability, angry rumination would be associated with later irritability over and above depressive rumination.

## Methods

### Design

Ethical (Institutional Review Board, IRB) approval for the study was granted by the King’s College London Psychiatry, Nursing and Midwifery Research Ethics Subcommittee (Reference: HR-15/16-1919). The study was performed in accordance with the ethical standards as laid down in the 1964 Declaration of Helsinki and its later amendments or comparable ethical standards. The study is part of a larger prospective project. The present study focused on two stages of classroom-based data collection over 6 months. At Time 1, demographics, irritability, and angry and depressive rumination were measured. At Time 2 (month 6), irritability was measured again.

### Recruitment and Sample

Data was collected from a non-selective secondary school that serves a culturally diverse community in London, UK (Ofsted 2013). The school has a higher proportion of black and ethnic minority students compared to the local borough. The local borough has a higher proportion of black and ethnic minority students compared to most other London boroughs and to the rest of England (London Borough of Lambeth 2018). The proportion of students eligible for free school meals, an index of deprivation (Taylor 2018), is very high (75%) (Ofsted 2013). All students in years 8 and 9 (aged 12 to 14 years) were invited to participate. Written informed young person assent and opt-out parental consent was sought to ensure a representative sample and maximise participation rates across time points. Data collection was carried out in schools, as part of a larger study. Researchers attended year assemblies to explain the project and hand information sheets to the students for themselves and their parents. Information sheets and opt-out parental consent forms were sent to parents by post and were also given to students to hand to their parents/carers. Parents/carers were given two weeks to opt-out on behalf of their child and were provided with three different methods for opting out: telephone, email and post (stamped, addressed return envelopes were provided). At both measurement points, researchers attended form periods and/or allocated humanities lesson and provided reminder information about the research project. Adolescents who agreed to participate signed assent forms. Those who did not wish to participate or who did not meet eligibility criteria were asked to read quietly. Young people signed opt-in assent forms at both time points and were free to withdraw at any point during the study.

There were 251 students on the school register for Years 8 and 9. Non-participation was due to absence on the day of testing, young-person non-assent, and parent opt-out. 165 students (94 (57%) male and 71 (43%) female) participated at Time 1. The average age was 13.22 years (SD = 0.63; Range = 12y 2 m-14y 5 m). 54.5% of adolescents identified their ethnicity as Black, 18.8% as White, 6.7% as Asian, 14.5% as Mixed and 4.8% as Other. The gender and ethnicity distribution of the final sample was representative of the school population. Of the students that participated at Time 1, 156 participated at Time 2. There was no significant association between gender (χ^2^(1) = 0.74, *p* = 0.39) or ethnicity (χ2(4) = 1.32, *p* = 0.86) and incompletion at Time 2.

### Measures

**Irritability** was measured with the Affective Reactivity Index (ARI; Stringaris et al. (2012)), a self-report questionnaire with 6 items, each ranging from 0 to 2 (total score: 0–12), including items such as ‘*I am easily annoyed by others*’. The ARI is an instrument that has been validated to measure irritability (Stringaris et al. 2012). There have been a number of studies that have demonstrated its associations with internalizing (Stoddard et al. 2014) and externalising symptoms (Humphreys et al. 2019) as well with behavioural correlates of irritability, such as aggression (Ezpeleta et al. 2020). The measure has been found to be reliable in healthy and clinical youth samples (Mulraney et al. 2014; Stringaris et al. 2012). Cronbach’s alpha for the ARI in the current study was 0.87 at Time 1 and 0.88 at Time 2.

**Depressive Rumination** was measured with the Rumination Subscale of the Children’s Response Styles Questionnaire (CRSQ; Abela et al. (2002)), total scores range from 0 to 39, and includes items such as ‘*When I am sad, I think “I’m ruining everything”.* The Rumination subscale of the CRSQ has been shown to predict onset of major depressive episodes over two years, controlling for baseline depressive symptoms and history of episodes (Abela and Hankin 2011), and predict increases in depressive symptoms at 6 week follow-up in children of parents with a history of depression (Abela et al. 2007). Adequate internal consistency has been demonstrated (Abela et al. 2004; 2007). Cronbach’s alpha for the rumination subscale in the current study was 0.92.

**Angry Rumination** was measured with the Children’s Anger Rumination Scale (CARS; Smith et al. (2016)) a 19-item measure with scores ranging from 19 to 76, including items such as ‘*When I am angry, I think a lot about other times when I was angry’*. The measure has been shown to be associated with peer and teacher rated aggression in a sample of male juvenile offenders and a sample of healthy adolescents (Smith et al. 2016). It has been shown to be reliable (Harmon et al. 2019; Smith et al. 2016). Cronbach’s alpha for the CARS in the current study was 0.94.

### Data Analysis

Descriptive statistics and intercorrelations were inspected, before undertaking three regression models. In all regression models, baseline irritability levels were entered first to test whether the independent variables predict prospective elevations in irritability over time. This provides a conservative and strong test of our hypotheses, because it accounts for possible overlap between symptoms and predictor variables (e.g. irritability and angry rumination), and also for the continuity of symptoms over time. Age and gender were also included at the first step. Then in the first two multiple linear regressions, we tested whether angry rumination (Model 1) and depressive rumination (Model 2) predict prospective irritability, by entering one of these rumination variables in the second step in each of the two models. Finally, in the third regression model, both rumination variables were added at the second step, to test the hypothesis that angry rumination makes an independent contribution to irritability, over and above depressive rumination (Model 3).

To determine the best-fitting model for the data, we compared both of the nested models (Models 1 and 2) to the more complex model (Model 3) using log-likelihood tests. Akaike’s Information Criteria (AIC) and Bayesian Information Criteria (BIC) statistics were used to evaluate model parsimony, with lower scores indicating a more parsimonious model.

## Results

### Preliminary Analysis

After data was entered, a random 10% was checked with double entry and analysis was undertaken in SPSS v.25 and R (R Core Team 2019). Mean substitution was performed when less than 5% of items were missing in each questionnaire. Questionnaires with more than 5% of missing items were treated as missing variables (data was complete for age, gender, and baseline irritability; *n* = 2 cases were missing the depressive rumination variable; *n* = 5 cases were missing the angry rumination variable; and *n* = 9 cases were missing outcome irritability). Little’s Missing Completely At Random (MCAR) test showed a non-significant result (*p* > 0.05), meaning that the data was MCAR. Missing data were accounted for using expectation maximization that included all variables entered in the regression models. Regression analysis was repeated with complete case analysis with no differences in findings.

Assumptions of multiple regression were met, namely: scatterplots indicated linear relationships between independent variables and the outcome variable; inspection of the Q-Q-Plot indicated multivariate normality; there was no evidence of multicollinearity based on pairwise correlations between predictor variables (all below 0.8; Berry et al. (1985)) and on variance inflation factor (VIF) values (all below 5; Neter et al. (1996)); finally, the scatterplot of residuals versus predicted values indicated that the assumption of homoscedasticity was met. Mahalanobis distance indicated two multivariate outliers. Analyses were run with and without these two cases, with no difference in results, so results with all 165 cases are presented. All variables, except for sex and age, were standardized.

### Descriptive Statistics

Descriptive statistics for the main variables are presented in Table 1. The mean irritability scores at baseline ($\overline{X}$=3.68 [SD = 3.21]) and outcome ($\overline{X}$=3.01 [SD = 2.96]) were within the range of scores observed in other studies with community samples. For example, a mean score of 4.00 (SD = 3.37) was reported in a community sample of Brazilian adolescents, and a mean score of 1.96 (SD = 2.25) was reported in a sample of unselected Australian adolescents ($\overline{X}$=1.96 [SD = 2.25]) (Mulraney et al. 2014). Mean depressive rumination scores ($\overline{X}$=10.82 [SD = 8.79]) were comparable to those observed in other samples of unselected youth; for example, a mean of 10.94 (SD = 7.65) in a large sample of US adolescents (McLaughlin and Nolen-Hoeksema 2012), and a mean of 14.78 (SD = 7.40) in a school-based study of 367 adolescents (Abela et al. 2009). The mean angry rumination score ($\overline{X}$=36.13 [SD = 12.96]) in the current study is comparable to that reported in the study of Harmon et al. (2019) ($\overline{X}$=39.61 [SD = 12.97]), in a sample of unselected US youth (average age: 10.61 [SD = 1.78]).

**Table 1:** Descriptive statistics and correlation matrix for main variables

	Total Sample Mean [SD]	Females Mean [SD]	Males Mean [SD]	Gender Difference *t*	1^†^	2^†^	3^†^	4^†^
Time 1								
Baseline Irritability	3.68 [3.21]	3.99 [3.00]	3.45 [3.34]	*t*(163) = 1.08	1			
Depressive Rumination	10.82 [8.79]	13.32 [9.14]	8.95 [8.07]	*t*(161) = 3.23**	0.38**	1		
Angry Rumination	36.13 [12.96]	37.20 [13.18]	35.33 [12.80]	*t*(158) = 0.90	0.64**	0.67**	1	
Time 2								
Outcome Irritability	3.01 [2.96]	3.39 [3.08]	2.72 [2.83]	*t*(193) = 1.57	0.62**	0.26**	0.50**	1

Significance levels (two-tailed): ** *p ≤ *0.01; * *p ≤ *0.05.

^†^ Correlations for the whole sample are presented, as no gender differences were observed.

Girls scored higher than boys in depressive rumination ($\overline{X}$(girls) = 13.32 [SD = 9.14] vs. $\overline{X}$(boys) = 10.82 [SD = 8.79]), but not in angry rumination ($\overline{X}$(girls) = 37.20 [SD = 13.18] vs. $\overline{X}$(boys) = 36.13 [SD = 12.96]). There were no other gender differences on the main variables.

As can be seen from the correlation matrix, putative predictors were significantly correlated with outcome irritability. As expected, there was a large correlation between angry rumination and outcome irritability and a small correlation between depressive rumination and outcome irritability (Cohen 1988). The correlation between angry and depressive rumination was large.

### Regression Analysis

Two multiple linear regressions examined the contribution of each rumination variable to later irritability. In Model 1 angry rumination was added to the baseline variables at the second step. The addition of angry rumination significantly improved the model (*F*(1, 160) = 5.31 (*p* < 0.05), explaining an additional 2% of the variance (total variance explained: 41.4%). As can be seen in Table 2, baseline irritability and angry rumination were significant predictors of later irritability. In Model 2, depressive rumination was added to the baseline variables at the second step. The addition of depressive rumination did not improve the model (*F*(1, 160) = 0.002 (*p* > 0.05)). As can be seen in Table 2, baseline irritability was the only significant predictor of later irritability.

**Table 2:** Regression models predicting outcome irritability

Model	Predictors	Dependent Variable: Irritability	
		β	*t*
1	Age	0.03	0.44
	Sex	0.05	0.81
	Baseline irritability	0.52	6.67 **
	Angry rumination	0.18	2.31 **
2	Age	0.03	0.55
	Sex	0.05	0.84
	Baseline irritability	0.63	9.60 **
	Depressive rumination	0.003	0.05
3	Age	0.03	0.45
	Sex	0.09	1.23
	Baseline irritability	0.50	6.51 **
	Angry rumination	0.29	2.90 **
	Depressive rumination	-0.15	-1.73

* *p* < 0.05; ** *p* < 0.01. β = standardised beta coefficient

Independent prospective associations between the rumination variables and irritability were then examined in another multiple regression (Model 3). Standardized betas are presented in Table 2. The addition of angry and depressive rumination to baseline variables in the second step improved the model (*F*(2, 159) = 4.24, *p* ≤ 0.05), although only a modest proportion of additional variance was explained (ΔR^2^ = 0.03). Angry rumination significantly predicted outcome irritability in addition to baseline irritability. Depressive rumination showed a negative, although non-significant, association with outcome irritability in this model. Although all VIF were below the suggested threshold of 5 for this model, it may be that this is a suppression effect due to the high correlation between angry and depressive rumination (*r* = 0.67). The final model was significant, (*F*(5, 159) = 24.77, *p* < 0.001), explaining a total of 42.0% of the variance.

### Model Comparison

A log likelihood test comparing Model 1 (baseline variables plus angry rumination) to Model 2 (baseline variables plus depressive rumination) indicated that Model 1 was a significantly better fit to the data compared to Model 2 ($\chi$ ^2^(1) = 5.39, *p* < 0.001). A log likelihood test comparing Model 1 to Model 3 (baseline variables plus both rumination variables) identified with no significant differences between the models ($\chi$ ^2^(1) = 3.09, *p* = 0.08). A log likelihood test comparing Model 2 with Model 3 showed that Model 3 was a significantly better fit to the data compared to Model 2 ($\chi$ ^2^(1) = 8.48, *p* < 0.001).

AIC values were 386.86 for Model 1, 392.24 for Model 2, and 385.76 for Model 3. BIC values were 405.49 for Model 1, 410.88 for Model 2, and 407.51 for Model 3. The AIC and BIC both indicated that Models 1 and 3 are more parsimonious than Model 2. Minimal differences in AIC and BIC were observed between Models 1 and 3. AIC indicated Model 3 is marginally more parsimonious than Model 1, whilst and BIC indicated the reverse. BIC penalises more heavily for more complex models and so taken together, the results indicate that Model 1, in which angry rumination is included in the model in addition to baseline variables, may provide the best and most parsimonious fit to the data.

### Examining Angry Rumination and Irritability Item Overlap

It is possible that the observed associations between angry rumination and irritability are not a result of an underlying association between the two constructs but instead an artefact caused by overlap in item content. For example, items such as “*I feel angry about certain things in my life*” and “*I keep thinking about events that angered me for a long time*” in the CARS angry rumination measure may overlap with items in the ARI irritability measure, such as “*I stay angry for a long time*”. We examined this possibility in additional analysis.

Previous factor analysis of the CARS (Smith et al. 2016; Sukhodolsky et al. 2001) has indicated a four factor model: ‘angry afterthoughts’, ‘thoughts of revenge’, ‘angry memories’, and ‘understanding of causes’. The ‘understanding of causes’ factor is comprised of four items: “*I think about the reasons people treat me badly*”, “*I have had times when I could not stop thinking about a particular conflict*”, “*I try to figure out what makes me angry*” and “*When someone makes me angry, I keep wondering why this happened to me*”. These items appear to show less overlap with ARI irritability items. We therefore reran the regression analysis of the optimal model (Model 1: baseline irritability, age, gender, and angry rumination) with this subscale of the CARS as the angry rumination predictor. Results are presented in Table 3. The results remained the same, with baseline irritability and the ‘understanding causes’ subscale of the angry rumination scale the only two independent predictors of later irritability. Although not presented here, results of regression model 3 were also the same when the ‘understanding causes’ subscale was used as the index of angry rumination.^1^

**Table 3:** Results of multiple regression with ‘understanding causes subscale’ of the angry rumination scale

Predictors	Dependent Variable: Irritability
	β
Age	0.06
Sex	0.09
Baseline irritability	0.56 **
Angry rumination – ‘understanding causes’ subscale	0.16 *

* *p* < 0.05; ** *p* < 0.01. β = standardised beta coefficient

## Discussion

In the present study, we examined rumination as a predictor of irritability in a community sample of adolescents. Angry rumination was associated with increasing irritability over time, whilst depressive rumination was not. Furthermore, angry rumination was associated with later elevations in irritability in adolescents, over and above depressive rumination.

We found that adolescents who tend to engage in depressive rumination are also more likely to engage in angry rumination. This is consistent with studies with adults (Peled and Moretti 2010), conduct disordered youth (Peled and Moretti 2007), and children (Harmon et al. 2019) and indicates that angry and depressive rumination are related constructs. For example, in the present study we observed a large correlation between angry and depressive rumination (r = 0.67), which falls between that reported in children (r = 0.56; Harmon et al. 2019), and in adults (r = 0.76; Peled and Moretti 2010). Findings are consistent with the hypothesis that whilst the two forms of rumination are related, they are distinct constructs. Specifically, angry and depressive rumination showed differential prospective associations with irritability. Whilst angry rumination was a significant predictor, depressive rumination was not. Then, when both forms of rumination were added to the regression model simultaneously, angry rumination was a significant independent positive predictor of later irritability, whilst depressive rumination was not. Again, this is consistent with cross-sectional findings of a unique association between angry rumination and angry feelings and aggressive behaviours in adult and child samples (Harmon et al. 2019; Peled and Moretti 2010).

An alternative explanation for our findings is that the observed differences in association between angry rumination and depressive rumination and irritability are due to item differences. In other words, it may be that differences in items (i.e. form) rather than focus (depression vs. anger) can account for the differences. However, Peled and Moretti (2010) used analogous scales (the only difference being words related to sadness and anger), to measure the two forms of rumination. They reported unique relations between each type of rumination and emotional and behavioural outcomes, suggesting they are distinct constructs.

Our findings are pertinent to understanding cognitive vulnerability factors for adolescent irritability. Research to date has indicated positive cross-sectional associations between angry rumination and angry feelings. Examination of Time 1 cross-sectional correlations in this study are of a similar magnitude to those reported elsewhere; for example, 0.64 with irritability in the current study, 0.50 with anger in the study of Peled and Moretti (2007). Extending beyond cross-sectional analysis, we observed that angry rumination at baseline was positively associated with outcome irritability six months later (*r* = 0.50). One possible account of these findings is that the association between angry rumination and subsequent irritability symptoms is due to the strong concurrent correlations between angry rumination and irritability scores. In other words, an adolescent may score highly on an angry rumination item such as ‘*I think a lot about other times when I was angry*’ because they are prone to this kind of thinking, or because they are irritable and so preoccupied with these thoughts at that time. However, we tested the prospective relationship whilst controlling for baseline levels of irritability, and the angry rumination – irritability association persisted. It therefore seems that a tendency to engage in angry rumination increases feelings of irritability in adolescents over time, rather than being due to a confound. We note that the increase in variance in outcome irritability explained with the addition of angry rumination is small (2–3%). However, small effects can accumulate over time (Funder and Ozer 2019); the tendency to engage in angry rumination may only modestly affect how irritated you feel, but this could accumulate fairly quickly over time, leading to more substantial effects in the long-term.

A further plausible explanation for the positive association between angry rumination and irritability is item overlap, by which the scales are correlated due to similarities in items rather than similarity in the underlying constructs. On inspection of the ARI irritability index and CARS measure of angry rumination, certain items of the two scales were indeed similar. We therefore reran the multiple regression analysis using a subscale of the CARS, ‘understanding causes’, as the index of angry rumination. This subscale was chosen because the constituent items showed less overlap with the ARI items. Findings of the multiple regression remained unchanged, which speaks against the suggestion that the observed associations are due to item overlap.

It is important to note a number of limitations of the study. All measures were self-report, and so replication of these results with multiple methods and multiple informants is needed. Participants in the present study were recruited from a community sample: whilst studies such as those of Stringaris and colleagues (Stringaris et al. 2012) support a dimensional view of irritability, and therefore indicate that findings regarding the associations between irritability and relevant constructs may be comparable across the severity spectrum, replication with a clinical sample will be important. Whilst the study was adequately powered to test the hypotheses of interest, the sample size was fairly modest, which limits closer examination of the associations of interest, such as the interactive effect of angry and depressive rumination, and the possible moderating role of gender. The use of a prospective design represents a strength of the study, however further studies measuring rumination at multiple time points and examining its temporal interplay with irritability, depression and their behavioural correlates will be valuable. For example, whilst the present study has demonstrated that angry rumination leads to increased irritability, it is very plausible that the association also operates the other way, with more irritable youth showing an increased tendency to engage in angry rumination over time.

The findings, should they be replicated in a clinical sample, highlight opportunities for delivering clinical benefit. Identifying psychological mechanisms that contribute to the persistence of irritability and can be modified has implications for the treatment of problematic irritability. For example, adolescents may be given psychoeducation about the unhelpful effects of angry rumination as an emotion regulation strategy, and encouraged to look out for warning signs and triggers for when it occurs. They may be trained to use more adaptive coping strategies, such as mindfulness (Wright et al. 2009), directed imagery, and active problem-solving (Watkins 2015) that they can then engage in as an alternative to rumination (Leigh et al. 2012).

## Footnotes

1 Results available on request from the corresponding author.
